# Calculation method of canopy effective volume for fruit tree based on LiDAR point cloud data

**DOI:** 10.3389/fpls.2025.1679027

**Published:** 2025-11-18

**Authors:** Hao Ma, Kexin Wang, Jingyuan Ma, Shijie Jiang, Pan Liu, Ce Yang, Dongdong Wang, Hongwei Cui, Haoyu Chang

**Affiliations:** 1 College of Agricultural Equipment Engineering, Henan University of Science and Technology, Luoyang, China; 2 Beijing Changping District Agricultural Technology Extension Station, Beijing, China; 3 Department of Bioproducts and Biosystems Engineering, University of Minnesota, Saint Paul, MN, United States

**Keywords:** fruit tree, canopy effective volume, LiDAR, improved alpha-shape algorithm, effective volume coefficient

## Abstract

The canopy volume of fruit trees is an important basis for precise orchard management. However, current methods for predicting canopy volume cannot accurately identify and exclude canopy porosity, resulting in a larger prediction than the actual volume. To address this issue, this study proposes a calculation method of canopy effective volume (EV) for fruit tree based on LiDAR point cloud data. In this method, the fruit tree canopy model is first reconstructed using an improved alpha-shape algorithm, and its volume is calculated. Then, the canopy effective volume coefficient was constructed, and the product of the two was used as the canopy effective volume. To evaluate the accuracy and applicability of the proposed method, both simulated fruit tree and orchard experiments were conducted and compared with the prediction results of alpha-shape by slices (ASBS), convex hull by slices (CHBS), and voxel-based (VB) methods. The results show that the best model prediction performance is achieved when the voxel size is the average nearest neighbor distance of the point cloud and the partition size is five times the voxel size. The method achieved an *R²* of 0.9720, an *RMSE* of 0.0203 m^3^, and an *MAE* of 0.0192. Compared with the prediction results of the ASBS, CHBS, and VB methods, the volume reduction rates were 0.5101, 0.6953, and 0.6213, respectively. The EV method can accurately quantify the canopy effective volume after removal of canopy porosity and provide decision support for precise orchard management.

## 1 Introduction

In the full cycle of orchard management, diseases and pests control constitutes approximately 30% of the overall workload. Spraying chemical pesticides is the primary method of controlling diseases and pests in orchards. However, under conventional spray methods, the actual pesticide utilization rate is less than 40%. It not only wastes resources but also poses a threat to the ecological environment and food safety (Guo et al., 2025; Meshram et al., 2022). In this background, the precise variable spray technology has become a significant breakthrough in sustainable diseases and pests management in orchards. The decision-making core of this technology relies on the precise sensing of fruit tree canopy characteristics (Salcedo et al., 2020). In fact, canopy volume serves as a key indicator of fruit tree growth status, and its precise measurement extends beyond variable spray. For example, the dynamic changes in canopy volume is an important basis for fruit tree yield estimation (Underwood et al., 2016). Precise three-dimensional canopy volume data provides the scientific basis for determining pruning levels and guiding precise pruning operations (Johansen et al., 2018). Therefore, precise measurement of canopy volume is crucial for achieving precision production management and enhancing quality and efficiency in orchards (Sun H. et al., 2024; Gu et al., 2021).

In the current study, the methods for calculating canopy volume based on LiDAR data include voxel-based (VB), convex hull (CH), alpha-shape (AS), convex hull by slicing (CHBS), and alpha-shape by slicing (ASBS) algorithms (Dong et al., 2024; Colaço et al., 2017; Gao et al., 2021; Sun D. et al., 2024). The VB algorithm works by discretizing the 3D point cloud space into a regular grid (voxels), counting the number of voxels occupied by the point cloud, and multiplying the volume of a single voxel to estimate the total volume. Bienert et al. (2014) proposed an improved VB algorithm for estimating canopy volume, which improves the problem of overestimation of canopy volume. However, the method’s accuracy is highly dependent on the voxel size; too large voxels can contain a large amount of porosity not occupied by the point cloud, resulting in a significant overestimation of the volume; too small voxels significantly increase the computational burden. More importantly, even for smaller voxels, the method essentially calculates the volume of the voxel containing the point cloud, rather than the actual canopy volume of the point cloud itself (Vonderach et al., 2012; Hosoi et al., 2013). Spaces within the voxel that are not filled by the point cloud, as well as the complex porosity structure within the canopy, are accounted for, which is the root cause of its large estimated volume. The CH algorithm estimates the volume of an object by connecting the outermost points and constructing the smallest convex polyhedron that encloses all the point clouds, forming a geometry without depressions. Cheein and Guivant (2014) used this algorithm to estimate the volume of tree tops from 3D laser data. Qi et al. (2021) applied the CH algorithm to calculate the volume of citrus canopy, achieving an *R²* of 0.8215 and an *RMSE* of 0.3186 m³. The main limitation of this algorithm lies in its “convexity constraint”, which forces the surface of the generated polyhedron to be convex outward. This constraint prevents it from accurately fitting the naturally occurring concave structures of the canopy. Additionally, it completely ignores the porosity structure within the canopy and considers the entire inner space of the convex hull as the canopy volume (Chakraborty et al., 2019). Therefore, the volume calculated by this method is generally significantly larger than the actual volume of the canopy. The AS algorithm is an optimized version of the CH algorithm, where the boundary tightness can be controlled by adjusting the *α* parameter. A smaller *α* allows the boundary to more closely follow the contours of the point cloud, preserving the concave structures, while a larger *α* results in a surface that approximates the CH algorithm. Hadas et al. (2017) combined the AS algorithm with principal components analysis and proposed a method for automatically estimating olive tree shape parameters using airborne laser scanning data. Wang et al. (2021) developed a mobile scanning system based on 3D simultaneous localization and mapping to acquire 3D data from orchards. The system calculates the height and canopy volume of fruit trees using point cloud statistical methods and the 3D AS algorithm. The surface generated by the AS algorithm also encloses a closed space containing all areas within the outer boundary defined by the canopy’s point cloud. However, like the CH algorithm, the AS algorithm does not differentiate between the internal porosity and the actual canopy (Wang et al., 2024), resulting in an overestimation of the actual canopy volume.

Considering that the CH and AS algorithms neglect the internal structure of the canopy, often resulting in overestimated volumes, they have been combined with slicing methods to improve reconstruction accuracy by better capturing internal canopy features (Siebers et al., 2024). The CHBS algorithm operates by vertically slicing the point cloud into layers, applying the CH algorithm to each layer independently, and summing the volumes of all layers to obtain the total canopy volume. Researchers have used this approach to estimate canopy volume and surface area, reducing computational errors associated with complex canopy structures (Xu et al., 2014; Fernández-Sarría et al., 2019). Although applying convex hulls in layers enables better fitting of external canopy contours in horizontal cross-sections, each layer still inherits the limitations of the CH algorithm. As a result, the total volume calculated remains significantly overestimated (Zhou et al., 2021). The ASBS algorithm follows a similar approach, but uses the AS algorithm for each layer of point cloud data. Yan et al. (2019) proposed a method using this algorithm to accurately compute the volume of a single tree canopy based on vehicle-mounted LiDAR data, mitigating the influence of porosity being mistaken for actual canopy volume. Dong et al. (2021) utilized the ASBS method to accurately estimate apple tree volume from UAV multi-view 3D data, achieving an MAPE of 8.07% and an RMSE of 0.55 m³, respectively. Compared to the CHBS algorithm, the ASBS algorithm more effectively captures the non-convex canopy boundaries in horizontal sections. However, for each layer, the AS algorithm calculates the volume of the area enclosed by the boundary of the point cloud for that layer, which contains the porosity within the boundary of that layer. When these layer volumes are summed, all intra-layer porosity is included. Furthermore, the algorithm is unable to identify and deduce which of the large canopy internal porosity run through multiple layers (Liu et al., 2021). Therefore, although the ASBS algorithm offers improvements over the CH or AS algorithms, it still inherently includes internal canopy porosity, leading to overestimated volume. The fundamental limitation of all methods mentioned above is that they compute the spatial envelope volume defined by the point cloud—essentially the minimum enclosed spatial extent of the point distribution—rather than the actual volume of the canopy. None of these approaches can effectively identify and remove the internal porosity of the canopy. As a result, the calculated volume includes all porosity between branches and leaves, leading to large predictions. If management decisions are based on this volume containing porosity, it will inevitably lead to issues such as over-spraying, yield prediction model distortion, and pruning plan inaccuracies, making it difficult to meet the demands of precision management in modern orchards (Wang et al., 2023; Liu et al., 2024; Huang et al., 2020; Liu et al., 2022).

In summary, in this study, to solve the problem of volume overestimation due to internal canopy porosity, a method for calculating the canopy effective volume of fruit tree based on LiDAR point cloud data was proposed. Canopy effective volume coefficient was developed to quantify the impact of canopy porosity. Orchard test was conducted, and compared and analyzed with current canopy volume prediction methods. The results of the study can effectively remove the influence of canopy porosity and provide reliable decision support for precise spray, yield estimation, and pruning management in orchards (Qiu et al., 2019; Walter et al., 2019).

## 2 Materials and methods

### 2.1 Experimental area

**Figure 1:**
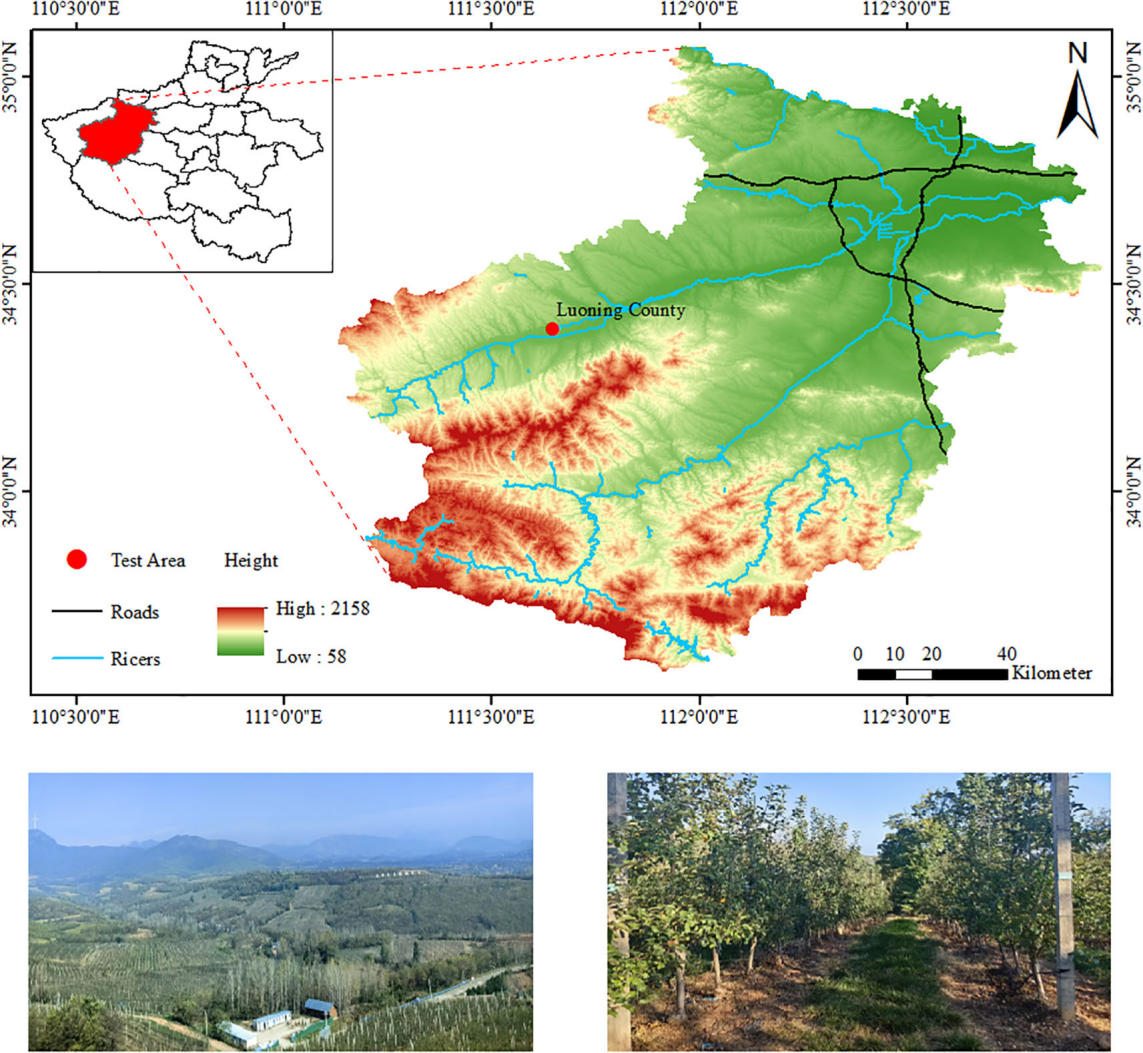
Experiment area.

The experiment was conducted in October 2024 in a standardized apple orchard in Luoning County (34°21′N, 111°15′E), Luoyang City. The experiment apple variety was Cripps Pink. The experiment orchard was planted in a tall spindle shape, with a row spacing of 3.5 m, plant spacing of 1.0 m, and an average tree height of approximately 3.5 m. The main trunk of the tree was erect and firm, with a spindle-like extension. Lateral branches were uniformly distributed around the trunk in a spiral layered arrangement, with branch angles ranging from 70° to 90°, forming a spindle-shaped canopy that was narrower at the top and broader at the base. The canopy between the rows was closely connected, forming a continuous “tree wall”. The canopy diameter of a single tree within a row ranged from 1.0 to 1.5 m. A total of 73 apple trees across three rows were selected as experimental subjects, and the experiment area and site layout are shown in **Figure 1**.

### 2.2 Point cloud acquisition equipment and methods

**Figure 2:**
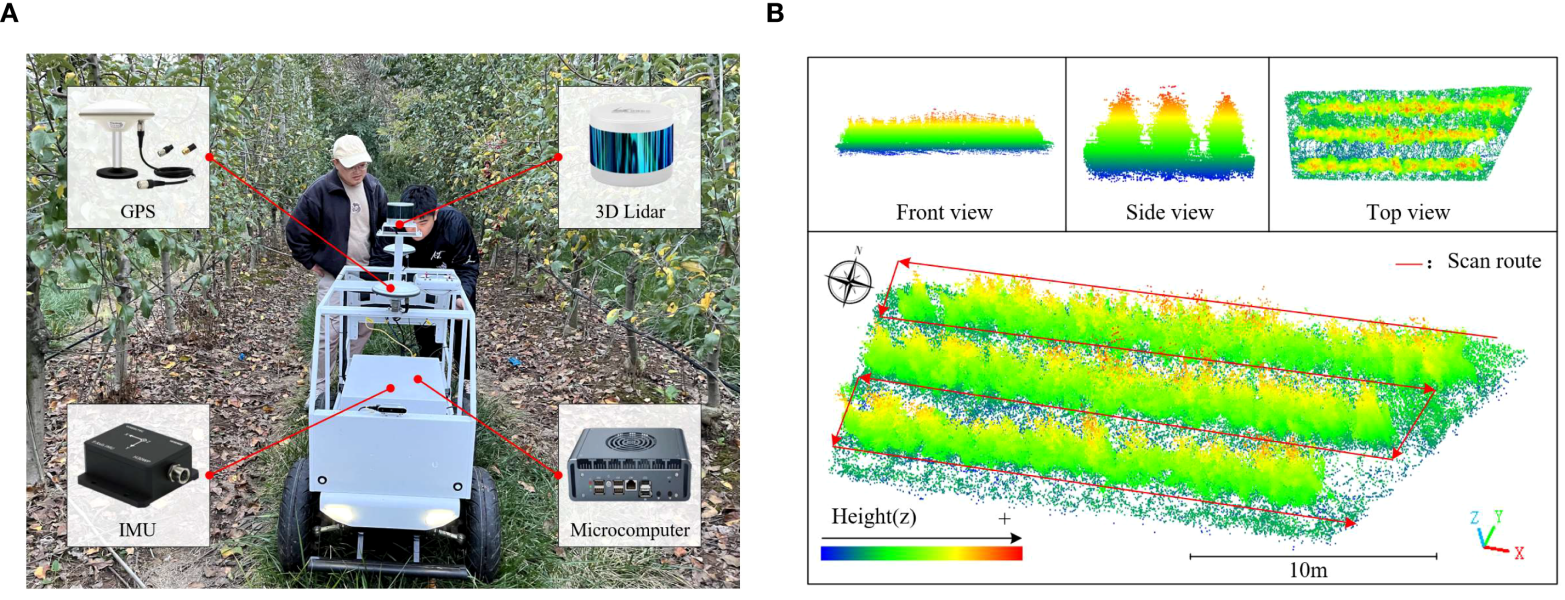
Point cloud acquisition equipment and the collected point cloud data. **(a)** An inspection trolley. **(b)** Point cloud data of the orchard experiment area.

In this study, an inspection trolley (Yuhesen, Shenzhen, China) was used to collect point cloud data of the fruit tree canopy. The inspection trolley comprises a mobile platform, a 3D LiDAR, an industrial computer, an IMU, and other components (**Figure 2a**). The mobile platform is the Yuhesen FR-07 Pro, powered by a 48V/20AH Li-ion battery, offering a range of 20 km. The 3D LiDAR has a range of 150 m, with a range accuracy of 2 cm, and a 16-wire harness. Its horizontal field of view (FOV) is 360°, and the vertical FOV spans from -15° to 15°. The depth camera has a resolution of 640x480 and a depth range of 0.2 to 4 m. The industrial computer is powered by an Intel Core i5-8265U CPU, with a base frequency of 1.6 GHz. The GNSS module can receive GPS signals, achieving a horizontal positioning accuracy of ±3 cm in RTK mode. The IMU has a measurement range of ±2000°/s, with a resolution of 0.01°/s.

The experimental part of this study included simulated fruit tree experiment and orchard experiment, and their point cloud data were collected respectively. In order to reduce errors, data acquisition was performed under clear, breezy conditions(wind speeds below 1.5 m/s). The experimenter controlled the inspection trolley to acquire the point cloud data in a closed loop around the experiment site line by line. **Figure 2b** shows the orchard experiment area, delineated using the point cloud cropping function in CloudCompare (v2.13.2) point cloud processing software. The data processing steps for both simulated fruit tree and orchard point clouds are consistent, and the subsequent processing is introduced as an example of orchard experiment area point cloud data.

### 2.3 Measurement method of canopy effective volume

**Figure 3:**
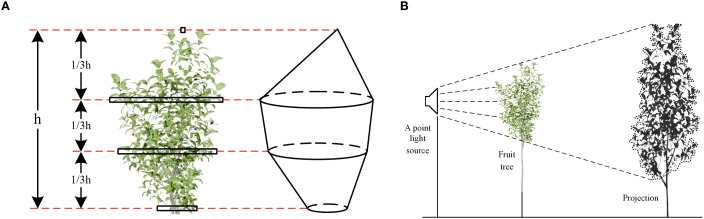
Manual measurement process of fruit tree canopy effective volume. **(a)** Manual measurement of canopy volume. **(b)** Measurement of effective volume percentage using the projection method.

The manual measurement of the canopy effective volume of fruit trees includes two main steps: firstly, measure the volume of the fruit tree canopy, and then use the projection method to measure the effective volume percentage. These two values are multiplied to obtain the canopy effective volume. When measuring the canopy volume, the fruit tree canopy was divided into three equal parts along the height: upper, middle, and lower sections (**Figure 3a**). The volume of the middle and lower sections was calculated using the circular table model, while the volume of the upper section was calculated using the conical model. The final canopy volume was the sum of the volumes of the three sections.

The schematic diagram for using the projection method to measure the effective volume percentage is shown in **Figure 3b**. A point light source and a projection screen were placed on both sides of the fruit tree. Under the illumination of the point light source, projection images of the canopy were sequentially captured from three orthogonal views: frontal, lateral, and top. An image processing method was then employed to calculate the ratio of the shaded area in each projection image to the total area of its outer contour. This ratio was defined as the effective coefficient in that projection plane. The plane effective coefficient quantifies the canopy’s effectiveness in that direction. However, projections in a single direction may misclassify porosity as effective volume due to occlusion. If it is porosity in another orthogonal direction, the effective coefficient for that direction will capture it. Therefore, the product of the effective coefficients in three orthogonal directions is used as the effective volume coefficient. This ensures that only canopy areas identified as effective in multiple directions are included in the final canopy volume. The multiplication rule ensures that porosity in any single direction reduces the overall effective volume coefficient, preventing misclassification due to occlusion. Additionally, to minimize measurement error, each fruit tree is measured three times, with a different projection angle selected for each measurement. The final result is the average of these three measurements.

### 2.4 Data preprocessing

#### 2.4.1 Removing noise points and ground filtering

**Figure 4:**
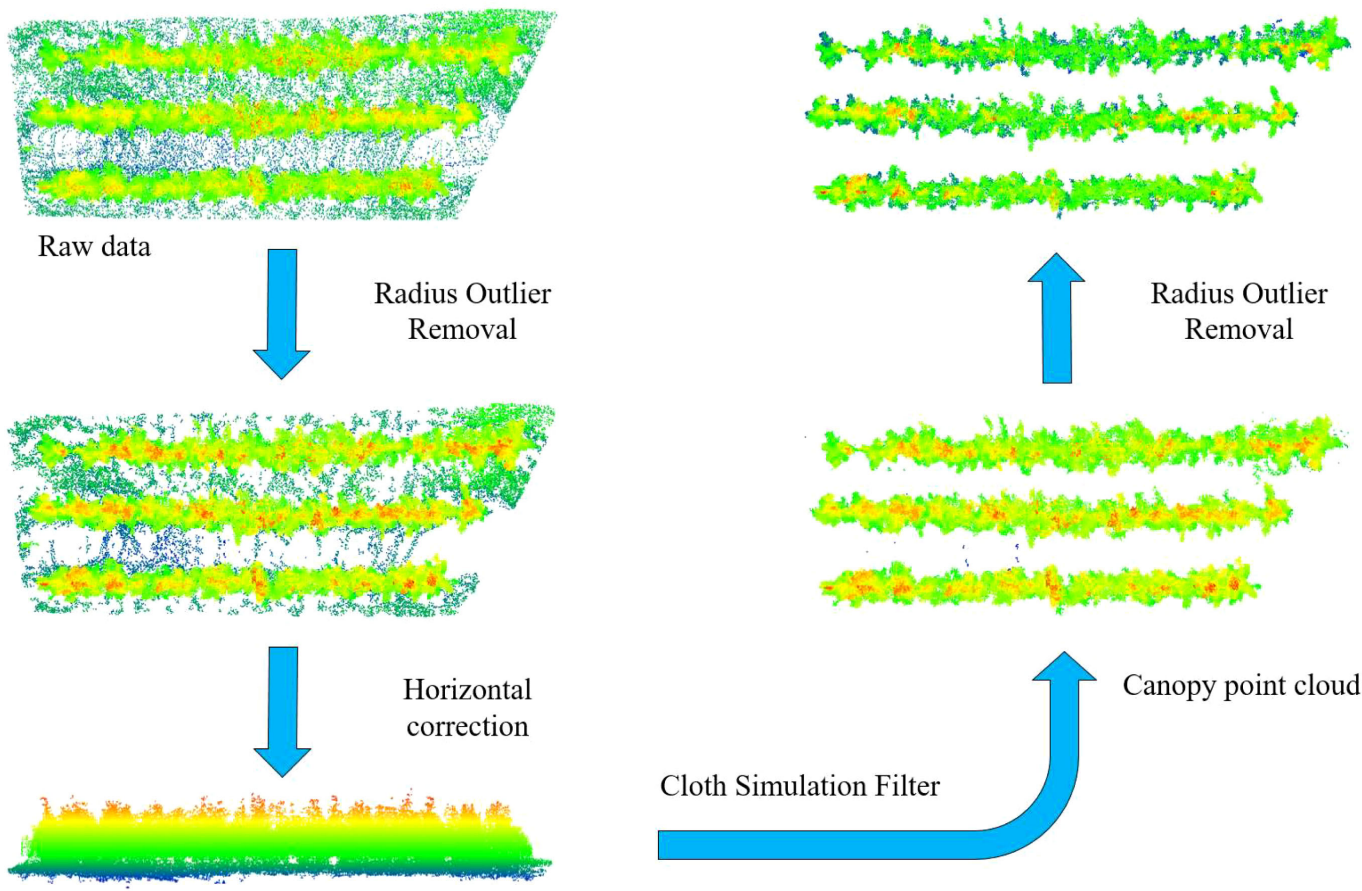
Workflow of point cloud preprocessing.

The raw point cloud data acquired from LiDAR scanning in the orchard often contains outlier noise points and ground points, which can result from sensor errors, environmental interference, and other factors. These noise points can negatively impact the calculation of the canopy effective volume of fruit trees, introducing errors in the results. In order to reduce the error, a four-step preprocessing procedure was developed for this study (**Figure 4**).

The Radius Outlier Removal (ROR) algorithm is used to eliminate outlier noise caused by sensor errors and environmental disturbances. This algorithm is a widely adopted denoising method in point cloud data processing (Cao et al., 2025). Its core principle is to remove points that do not meet predefined criteria by analyzing the point density in the neighborhood of each point. Compared to other noise reduction algorithms, this method is computationally simple, efficient, and suitable for processing large-scale point cloud data. Moreover, it effectively removes noise while preserving the overall structure of the point cloud, thus preventing excessive smoothing that could lead to the loss of important details. In this study, the search radius was set to 0.2 m, and the minimum number of neighborhood points threshold was set to 20.

During the traveling of the inspection trolley, ground bumps, undulations, and other factors can lead to a certain slope in the acquired point cloud relative to the actual scene. This is not conducive to the subsequent removal of the ground points, so it is necessary to calibrate the horizontal plane. The ground plane is roughly extracted using a random sampling consistency algorithm, and the normal vector of the fitted plane is calculated. The point cloud was then rotated to align the normal vector with the Z-axis.

After the horizontal plane is calibrated, the ground point cloud is separated using the Cloth Simulation Filtering (CSF) algorithm, which is a point cloud filtering method based on physical simulation. The core idea of this algorithm is to simulate a virtual fabric covering the inverted terrain surface under the effect of gravity, distinguishing between the ground and non-ground features through the settlement process of the fabric nodes. Compared with other ground filtering algorithms, the CSF algorithm can be adapted to various complex terrains without the need for a preset model and has high noise immunity (Li et al., 2024). In this study, the CSF package in Python was used to implement ground point removal. The algorithm involves six key parameters. The BSloopSmooth parameter determines whether to smooth the slope of the fabric nodes. Enabling this parameter for terrains with slopes greater than 30° significantly reduces misclassification. The class_threshold parameter sets a threshold for the maximum distance from the point cloud to the fabric nodes, with points within this threshold classified as ground points. The class_resolution parameter controls the cell size of the fabric grid; smaller values result in a finer map model. The iterations parameter determines the maximum number of iterations for fabric settling, with higher values improving precision but increasing computation time. The rigidity parameter reflects the fabric’s resistance to deformation, and a lower value makes the fabric softer, which better adapts to rugged terrain, but requires more iterations and slower settling speed. Lastly, the time_step parameter controls the time increment of the simulated physical process, which in turn affects the settling speed. The values of the parameters in this study are False, 0.3, 0.1, 500, 3, and 0.65, respectively.

After filtering with the CSF algorithm, some scattered weeds or ground points may remain and not be fully removed. To address this, we again used the ROR algorithm to eliminate local outlier points and provide accurate fruit tree canopy point cloud data for canopy effective volume calculation.

#### 2.4.2 Dividing the area for 3D reconstruction

**Figure 5:**
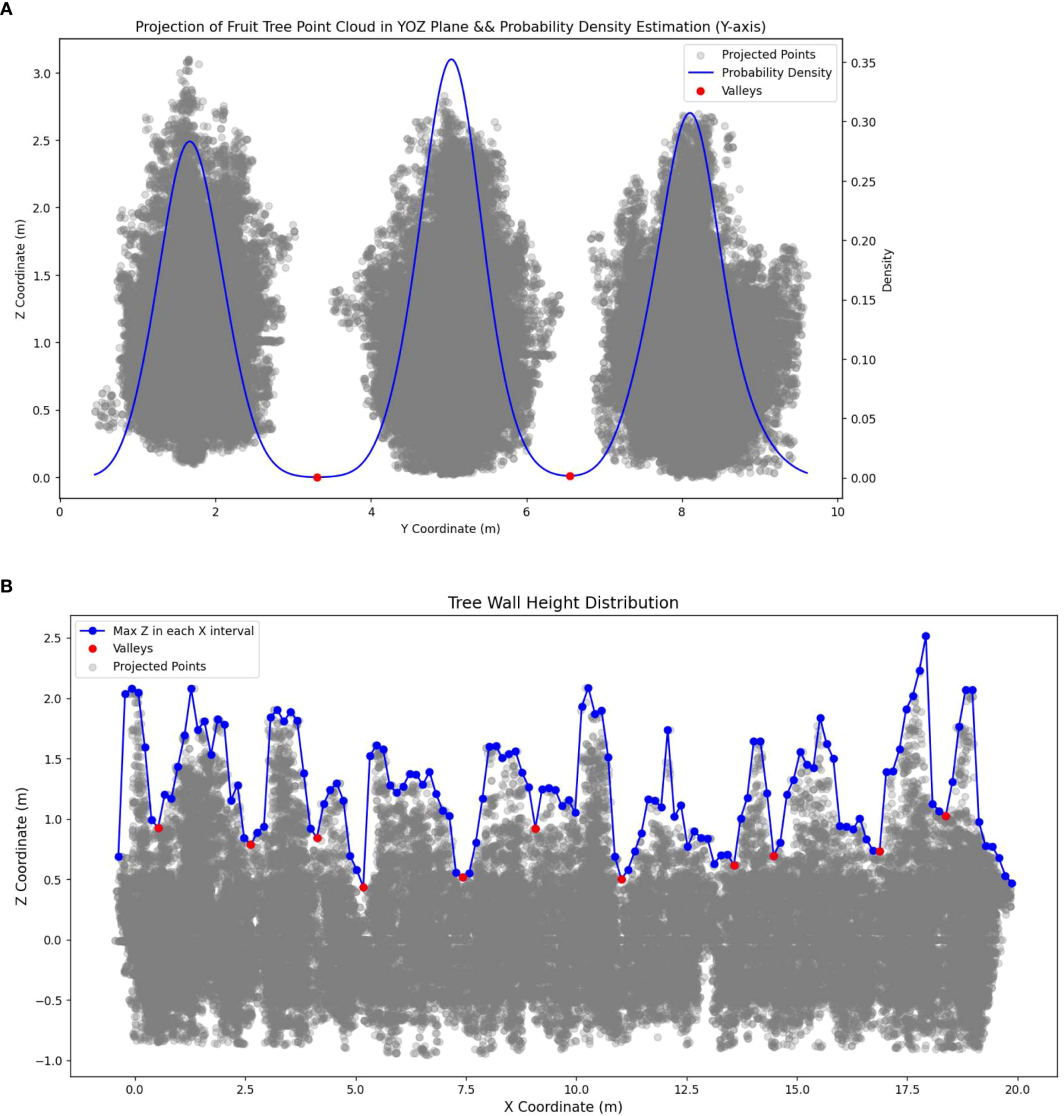
Schematic diagram of 3D reconstruction area division. **(a)** Fruit tree Y coordinate probability density distribution and row divide point. **(b)** Tree wall height distribution and column divide points.

To ensure the accuracy of the 3D reconstruction of the fruit tree canopy, it is necessary to perform 3D reconstruction area segmentation of the fruit tree canopy point cloud data. The first step is to segment the rows of fruit trees and extract the 3D point cloud data for each row within the study area. Since the experimental orchard is planted in a standardized manner with neatly arranged rows and columns of fruit trees, a row detection method based on probability density estimation of point cloud coordinates was used for segmentation. The probability density distribution of Y coordinates was calculated using Gaussian kernel density estimation, and fruit tree row segmentation was performed based on the trough locations and boundary points in this distribution (**Figure 5a**).

The second step was to divide the fruit trees into columns. However, due to the tall spindle planting in the experimental orchard, the canopies between adjacent trees are closely connected, forming a continuous “tree wall,” which makes it difficult to segment individual fruit trees effectively. Additionally, directly reconstructing the entire row of fruit trees yields significant errors. To address this, a method is proposed in this study to divide the 3D reconstruction area based on the distribution of height troughs within the tree wall. The specific process is as follows:

- Height distribution extraction: The point cloud data is divided into multiple partitions along the X-axis. In each partition, the maximum Z-value (that is, the highest point of the canopy) is computed, and a sequence of maximum Z-values is formed based on the order of the X-axis partitions.
- Trough detection: The troughs in the sequence of maximum Z-values are identified.
- Valid trough screening: A threshold is set for the minimum Z-value difference between a trough and its adjacent peaks. If the Z-value difference between a trough and any of its neighboring peaks is less than this threshold, it is classified as a pseudo-trough and removed. This step filters out invalid troughs with smooth height changes.
- Trough spacing constraint: A horizontal distance threshold is set between adjacent troughs. If the distance between two troughs is less than the threshold, the trough with the lower Z-value is retained. This ensures that the segmentation regions maintain a reasonable horizontal scale.
- Supplementary significant trough: A Z-value difference threshold is set between a trough and its neighboring peaks. If this difference exceeds the threshold, the trough is considered significant and retained. This step is intended to supplement the valid troughs with substantial differences in height that may have been removed during step 4.
- Segmentation point determination and column segmentation: All troughs retained after steps 3, 4, and 5 are merged and used as the final segmentation points for dividing fruit tree columns. Based on these points, fruit tree column segmentation is completed (**Figure 5b**).

### 2.5 Calculation of canopy effective volume of fruit trees

First, the point cloud data of the fruit trees were reconstructed using the AS algorithm with dynamic parameter optimization, and the volume of the reconstructed model was calculated as *V_m_*. Then, to quantify the proportion of the actual canopy volume within the reconstructed model volume, an effective volume coefficient *EVC* was introduced. Finally, the canopy effective volume (*V_e_*) was defined as the product of the reconstructed model volume (*V_m_*) and the effective volume coefficient (*EVC*). The mathematical expression is as follows:

(1) $$V_{e}=V_{m}\cdot EVC$$

#### 2.5.1 Canopy reconstruction and reconstruction model volume calculation

The AS algorithm is a surface reconstruction method based on computational geometry, capable of extracting 3D surface models with complex topology from discrete point cloud data. The core idea is to control the fineness of surface details through an adjustable parameter *α*. However, when *α* is too small, the reconstruction tends to include more detailed features but may result in surface fragmentation. Conversely, when *α* is too large, the reconstructed surface appears overly smooth and loses fine details. Therefore, selecting an appropriate *α* value is critical. However, the traditional AS algorithm uses a globally fixed *α* value, which is difficult to adapt to the density inhomogeneity of fruit tree canopy point cloud data.

Therefore, this study proposes an AS algorithm based on dynamic parameter optimization. By introducing a parameter-adaptive adjustment mechanism, the applicability and reconstruction quality of the AS algorithm in complex canopy scenarios are significantly enhanced. Specifically, smaller *α* values are selected in dense regions of the point cloud to preserve details, while larger *α* values are used in sparse regions to prevent surface fragmentation. This adaptive adjustment mechanism is implemented through a density-aware baseline α calculation combined with an iteratively optimized *α* incremental strategy.

Define a baseline *α* value based on the average nearest neighbor distance of the point cloud, calculated as shown in Equation 2:

(2) $$a_{base}=k\times d_{avg}$$

Where *d_avg_* is the average point distance, *k* is an empirical scaling factor initially set to 5.

The goal of the iterative optimization strategy is to gradually increase the value of *α* starting from the baseline value until a closed mesh is generated. The mathematical description of this process is shown in Equation 3:

(3) $$\left\{ \begin{array}{l} a_{i+1}=a_{i}+\Delta a\\ a_{i=0}=a_{base}\\ \Delta a=a_{step}\times d_{avg} \end{array}\right.$$

Where *α_i+1_* is the value of *α* for this iteration, *α_i_* is the value from the previous iteration, *Δα* is the increment of each iteration, and *α_step_* is the step coefficient that controls the magnitude of adjustment, set to 0.5 in this study.

**Figure 6:**
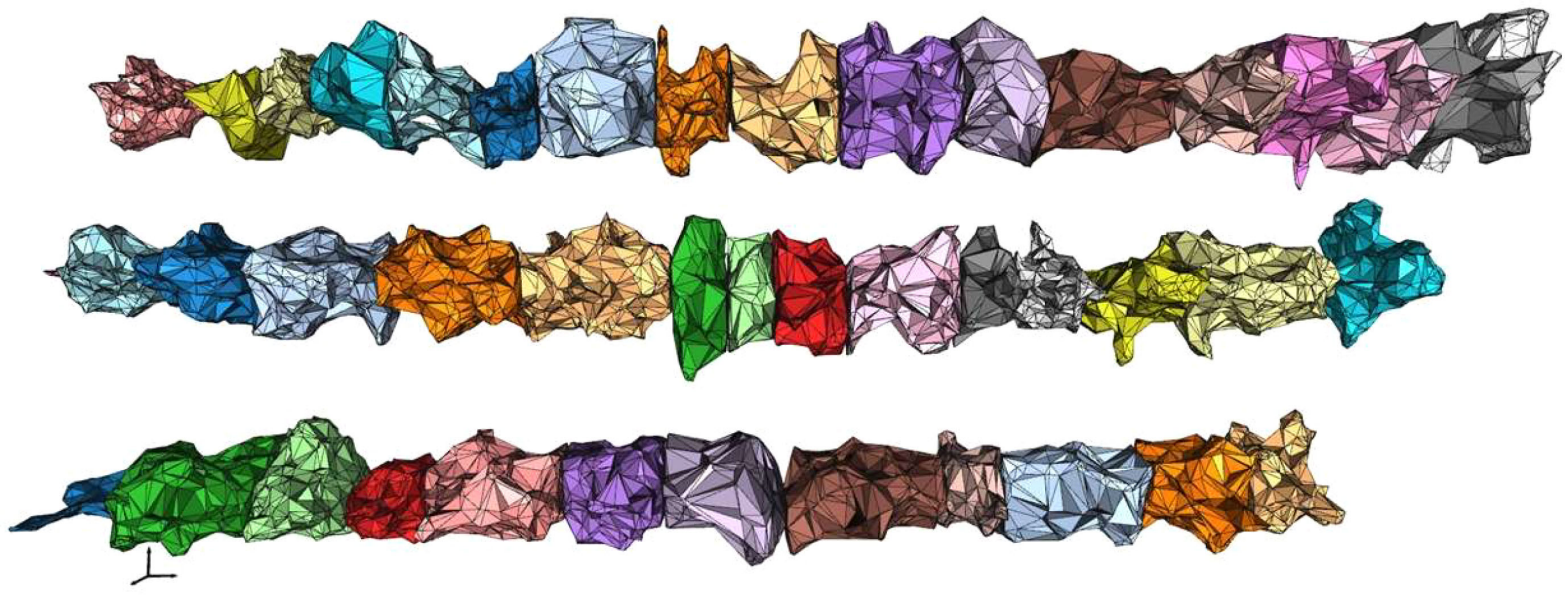
3D reconstruction canopy model of fruit trees.

When applying this method to canopy reconstruction, an appropriate *α* value is first calculated for the designated 3D reconstruction area. Surface reconstruction is then performed using this *α* value (**Figure 6**). Finally, the volume *V_m_* of the closed mesh of the reconstructed model is calculated as follows:

(4) $$V_{m}=\sum_{n=1}^{N_{g}}\left(\frac{1}{6}|{\vec{V}}_{0}\cdot (\vec{V_{1}}\times \vec{V_{2}})|\right)$$

Where *N_g_* is the number of triangle meshes in the reconstructed model, *V_0_*, *V_1_*, and *V_2_* are the vertex vectors of each triangle in the mesh, respectively.

#### 2.5.2 Calculation of effective volume coefficient

**Figure 7:**
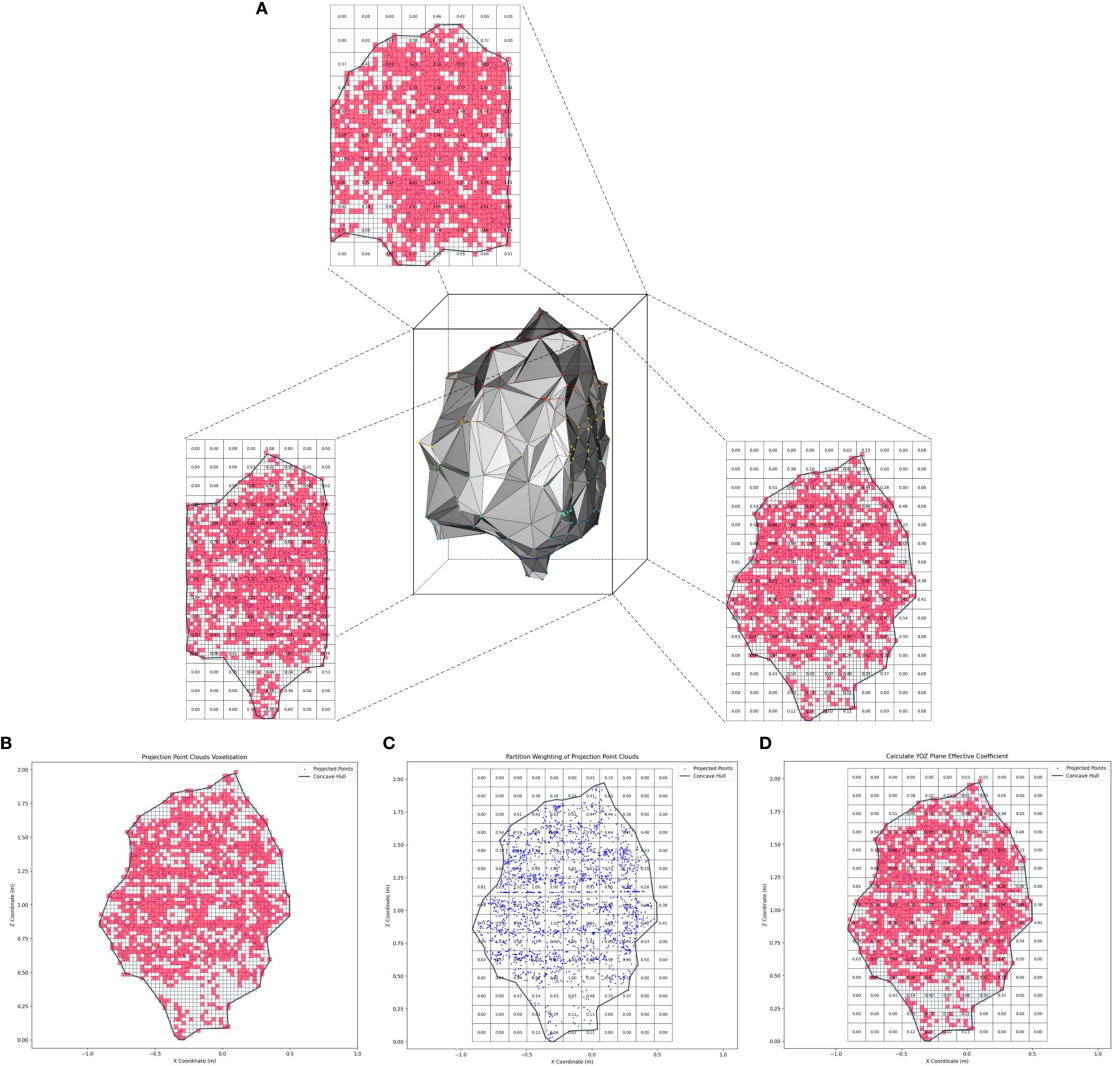
Schematic diagram of the effective volume coefficient calculation. **(a)** Three orthogonal projection planes for fruit tree canopy point clouds. **(b)** Projected point cloud voxelization. **(c)** Projected point cloud partitioning and weighting. **(d)** Schematic diagram of the calculation of the plane effective coefficient.

The effective volume coefficient *EVC* constructed in this study consists of the effective coefficients of the fruit tree canopy point cloud in the three orthogonal projection planes xoy, xoz, and yoz (**Figure 7a**), calculated as follows:

(5) $$EVC=EC_{xoz}\times EC_{xoy}\times EC_{yoz}$$

Where *EC_xoy_*, *EC_xoz_*, and *EC_yoz_* represent the effective coefficients of the xoy, xoz, and yoz projection planes, respectively.

The effective coefficients *EC_xoy_*, *EC_xoz_*, and *EC_yoz_* are calculated using a similar approach. In this study, the yoz projection plane is used as an example to illustrate the calculation of *EC_yoz_*.

(1) Projected point cloud voxelization.

First, the 3D point cloud data of the fruit tree canopy was projected onto the *yoz* plane. This dimensionality reduction was achieved by ignoring the *x*-axis coordinates while preserving the spatial distribution characteristics of the point cloud in the horizontal (*y*) and vertical (*z*) directions. Next, the projected point cloud was voxelized to discretize the data spatially. During the voxelization process, the *yoz* plane was divided into uniform square voxels, and the voxels containing projected point cloud data were marked as effective voxels (red), forming a spatial representation of the discretized point clouds (**Figure 7b**). In this study, the average nearest neighbor distance of the projected point cloud was used as the optimal voxel size, *VS*, a key parameter for voxelization. Finally, the AS algorithm was used to reconstruct the 2D boundary contour of the projected point cloud and extract all voxels enclosed within this contour.

(2) Projected point cloud partition weighting.

Square grids with sides equal to five times the voxel size (*VS*) were used to partition the projected point cloud (**Figure 7c**). This grid size was designed to ensure that the voxels generated in the previous step could be fully encompassed within each partition. For each partition, the polar deviation in the x-axis direction of the projected point cloud it contains, that is the canopy thickness of the canopy reconstructed model in that partition, was calculated as the weight W of the partition. The calculation method is shown in Equation 6:

(6) $$W=x_{max}-x_{min}$$

Where *W* is the partition weight, *x_max_* is the maximum x-coordinate value of the point cloud within the partition, and *x_min_* is the minimum.

During the weight calculation process, some partitions within the projected point cloud contour may have a weight value of zero because the point cloud data is absent. This contradicts the physical meaning that the reconstructed canopy model should have a non-zero thickness within the contour-enclosed partitions. To address this, an iterative interpolation strategy based on eight neighborhood averaging was proposed. For each zero-weight partition, its eight surrounding neighboring partitions were retrieved, and the arithmetic mean of the non-zero weight partitions was computed and assigned as the current partition weight. This process was iteratively repeated until all zero-weight partitions within the contour were eliminated.

(3) Calculate the effective coefficient of the projection plane.

All voxels contained within the boundary contour of the projected point cloud were mapped to the corresponding partitions (**Figure 7d**). For each partition, the total number of voxels (*n_av_*) and the number of effective voxels (*n_ev_*) were computed. The projection plane effective coefficient (*EC_yoz_*) is the ratio of the cumulative sum of the product of the number of effective voxels (*n_ev_*) in all partitions and the partition weight *W* to the cumulative sum of the product of the total number of voxels (*n_av_*) in all partitions and the partition weight *W*. The mathematical expression is as follows:

(7) $$EC_{yoz}=\frac{\sum_{i=1}^{N_{yoz}}W_{iyoz}\times n_{ievyoz}}{\sum_{i=1}^{N_{yoz}}W_{iyoz}\times n_{iavyoz}}$$

Where *n_yoz_* is the total number of partitions in the yoz projection plane, *W_iyoz_* is the weight of the ith partition, *n_ievyoz_* is the number of effective voxels in the ith partition, and *n_iavyoz_* is the total number of voxels in the ith partition.

Following the same approach, *EC_xoy_* and *EC_xoz_* can be computed as:

(8) $$\left\{ \begin{array}{l} EC_{xoy}=\frac{\sum_{i=1}^{N_{xoy}}W_{ixoy}\times n_{ievxoy}}{\sum_{i=1}^{N_{xoy}}W_{ixoy}\times n_{iavxoy}}\\ EC_{xoz}=\frac{\sum_{i=1}^{N_{xoz}}W_{ixoz}\times n_{ievxoz}}{\sum_{i=1}^{N_{xoz}}W_{ixoz}\times n_{iavxoz}} \end{array}\right.$$

In summary, combining Equations 1, 4, 5, 7, and 8, the mathematical expression for the method of calculating the canopy effective volume of fruit trees is:

(9) $$\begin{array}{c} V_{e}=\sum_{n=1}^{N_{g}}\left(\frac{1}{6}|{\vec{V}}_{0}\cdot (\vec{V_{1}}\times \vec{V_{2}})|\right)\times (\frac{\sum_{i=1}^{N_{xoy}}W_{ixoy}\times n_{ievxoy}}{\sum_{i=1}^{N_{xoy}}W_{ixoy}\times n_{iavxoy}}\\ \times \frac{\sum_{i=1}^{N_{xoz}}W_{ixoz}\times n_{ievxoz}}{\sum_{i=1}^{N_{xoz}}W_{ixoz}\times n_{iavxoz}}\times \frac{\sum_{i=1}^{N_{yoz}}W_{iyoz}\times n_{ievyoz}}{\sum_{i=1}^{N_{yoz}}W_{iyoz}\times n_{iavyoz}}) \end{array}$$

### 2.6 Calculation model construction of canopy effective volume of fruit trees

**Figure 8:**
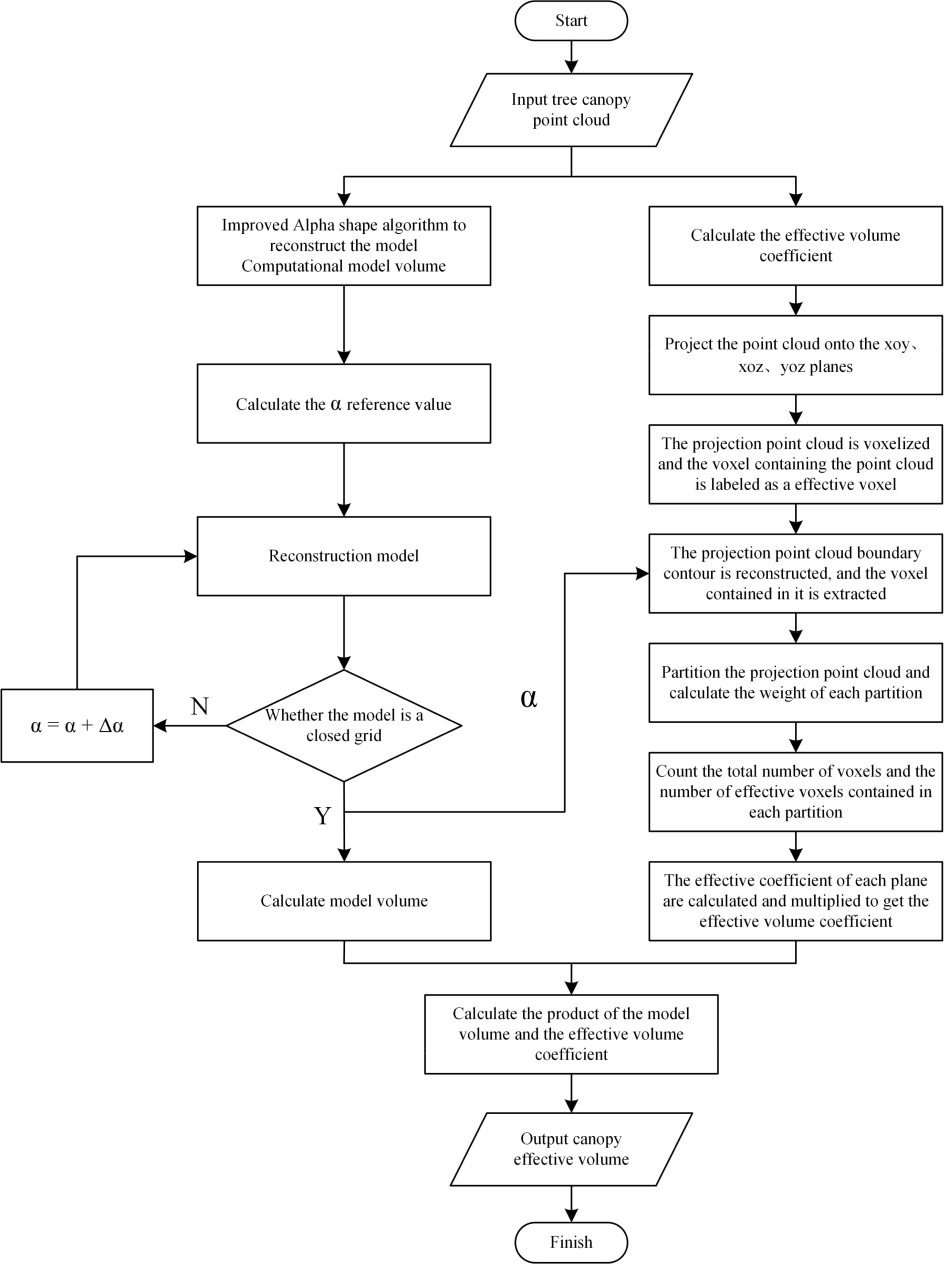
Workflow of the fruit tree canopy effective volume calculation model.

The canopy effective volume calculation method proposed in this study for fruit trees has been implemented as an automated computational model, which transforms the theoretical model into an executable program through a modular architecture. The model is developed in Python and integrates several key libraries, including the Open3D point cloud processing library, the NumPy scientific computing library, the alphashape library for boundary computation of point sets, the shapely.geometry library for planar geometric object processing, and the matplotlib library for scientific visualization. Together, these tools support a complete computational workflow from the input of fruit tree canopy point cloud data to the output of the final canopy effective volume. The overall computational flow of the model is illustrated in **Figure 8**. The core workflow is as follows:

- Data input: Load fruit tree canopy point cloud data.
- Dynamic reconstruction and volume calculation: The Alpha Shape algorithm with dynamic parameter optimization is used for 3D reconstruction of the canopy. The algorithm calculates the baseline α value through the density-aware mechanism and dynamically adjusts the parameters based on the iterative optimization strategy until a closed grid is generated. After reconstruction, the reconstructed model volume *V_m_* is computed (Equation 4).
- Calculation of effective volume coefficient: The system projects the point cloud to the xoy, xoz, and yoz planes for parallel processing. Each projection plane performs voxelization, partition weighting, and weight optimization. The effective coefficients for each plane are then computed based on the weighted effective ratio (Equation 7) and synthesized into a 3D effective volume coefficient *EVC* (Equation 5).
- Result output: The model automatically calculates the fruit tree canopy effective volume *V_e_* (Equation 9) and outputs the result.

### 2.7 Experimental validation design

In orchards, the canopy effective volume of fruit trees cannot be measured using the projection method due to orchard planting patterns and field conditions. However, simulated fruit trees can measure canopy effective volume in the laboratory. Therefore, this study conducted both a simulated fruit tree experiment and an orchard experiment. The simulated fruit tree experiment was used to verify the feasibility and accuracy of the method and to evaluate its effectiveness in removing canopy porosity by comparing it with current approaches. The orchard experiment was conducted to assess the applicability of the method in complex environments.

**Figure 9:**
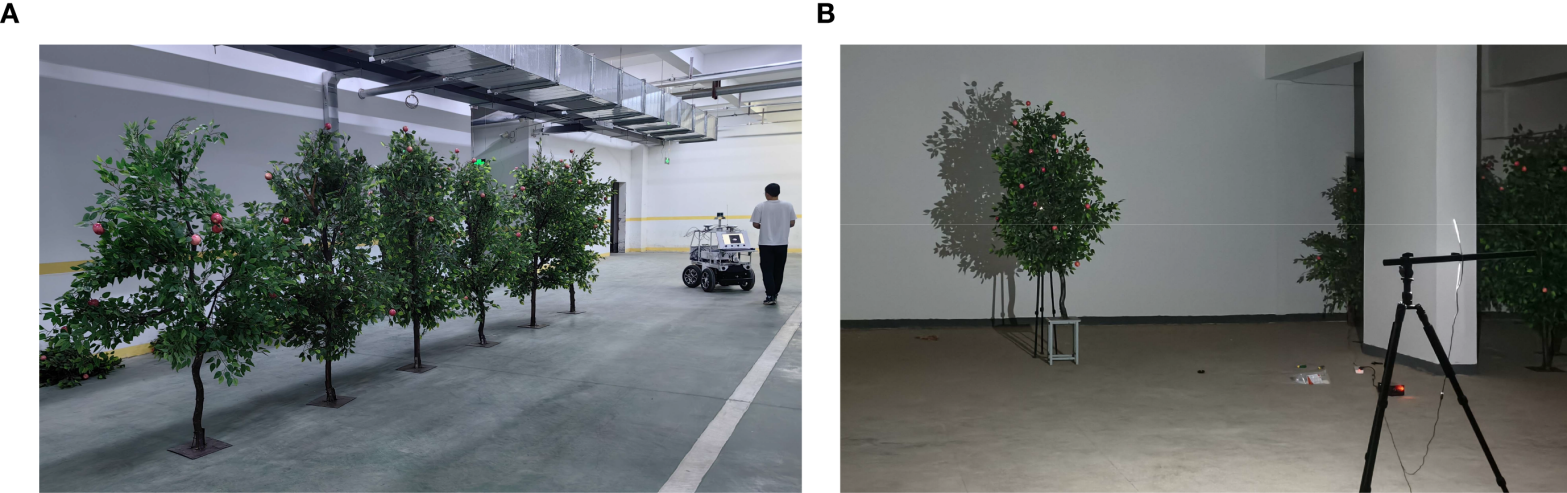
Simulated fruit tree experiment. **(a)** Point cloud data acquisition. **(b)** Projection-based measurement method.

Six simulated fruit trees with removable branches were used as experiment subjects to simulate canopy volume changes by varying the number of branches. Each simulated fruit tree was designed with four different branch combinations, and the experiment was repeated 4 times, resulting in a total of 24 tree samples. The experimental procedure included the following steps: (1) operating the inspection trolley to acquire point cloud data (**Figure 9a**); (2) measuring canopy effective volume in the laboratory environment (**Figure 9b**); (3) using the EV method to predict the canopy effective volume, and evaluating its performance through the metrics of the coefficient of determination (*R^2^*), the root mean square error (*RMSE*), and the mean absolute error (*MAE*); and (4) comparing the prediction results with those obtained from the ASBS, CHBS, and VB method, while quantifying the porosity removal effect using the volume reduction rate. The volume reduction rate can be calculated using Equation 10:

(10) $$VRR=\frac{V_{\text{c}m}-V_{EV}}{V_{cm}}$$

Where *VRR* is volume reduction ratio, *V_cm_* is the calculation result of the current method, and *V_EV_* is the calculation result of the EV method.

During the experiment, the number of slices used in both the ASBS and CHBS algorithms was set to 10; that is, the canopy point cloud data were divided into 10 equal layers along the Z-axis for volume calculation (Yan et al., 2019). The voxel size parameter of the VB algorithm was set to 1/15 of the canopy diameter (Lecigne et al., 2018).

The collected orchard point cloud data also employed the EV, ASBS, CHBS, and VB methods to predict canopy volume. The volume reduction rate of the EV method was then calculated relative to the other methods. The results were compared with those obtained in the simulated fruit tree experiment to determine whether consistent trends were observed, thereby assessing the stability and applicability of the EV method in complex orchard environments.

## 3 Results

### 3.1 Simulated fruit tree experiment results and analysis

#### 3.1.1 EV algorithm predicted results compared to measured values

**Figure 10:**
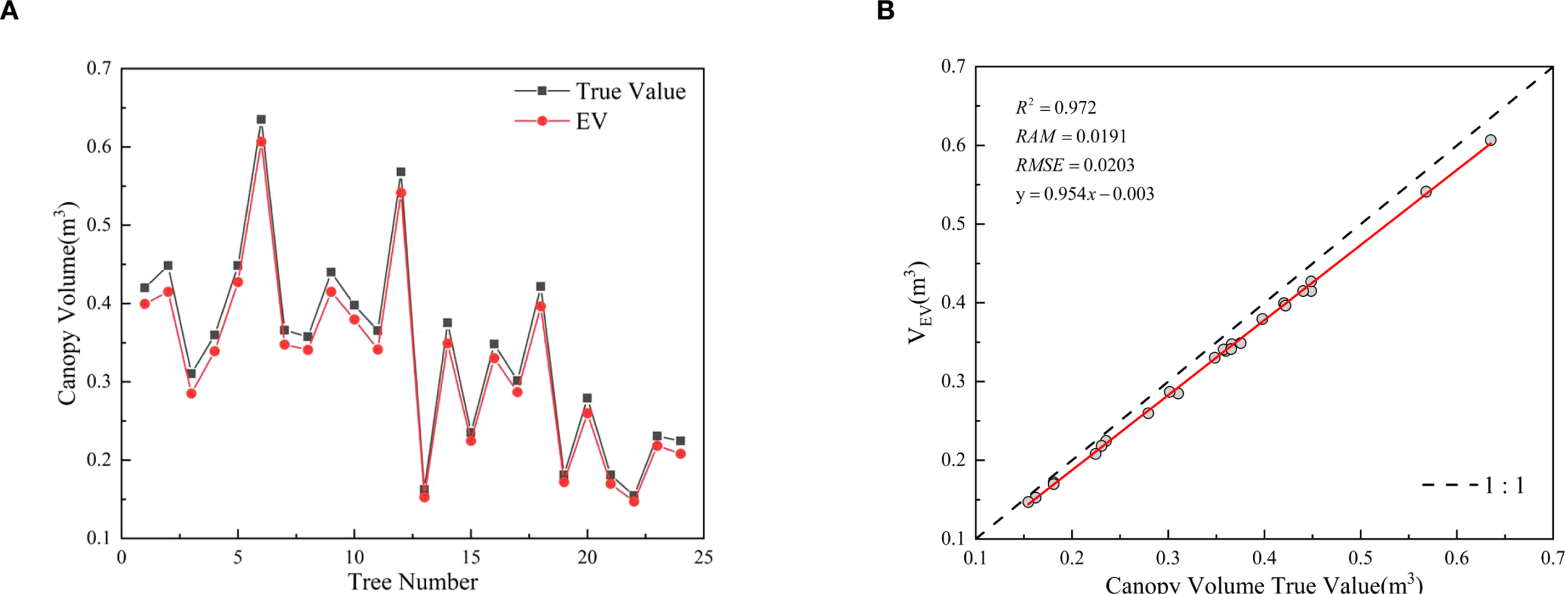
EV method predictions and canopy effective volume measurements.

The predicted values of canopy effective volume calculated by the EV method were analyzed in comparison with the manual measurements (**Figure 10a**). In the figure, black lines represent the measured values, while red lines denote the predicted values from the model. The results demonstrate that the proposed method can accurately predict the canopy effective volume of fruit trees with varying canopy sizes, showing a high degree of consistency between the predicted and measured values. Evaluation metrics for the EV method’s prediction performance: *R^2^* is 0.9720, *RMSE* is 0.0203 m^3^, and *MAE* is 0.0191. These evaluation metrics fully confirm that this method achieves high accuracy in predicting the canopy effective volume of fruit trees.

**Figure 10b** showed that the measured values were generally slightly higher than the predicted values. This phenomenon can be attributed to the measurement principle of the projection method, which utilizes a point light source to illuminate the fruit tree canopy, thereby generating a projection. The divergent nature of the light leads to an amplification effect, resulting in a projection size larger than the actual size of the canopy. Specifically, the projected size of the leaf near the light source side is larger than the leaf itself when projected onto the rear canopy, thus obscuring some of the rear porosity. This results in a high calculation of the effective coefficient, which ultimately leads to a systematic overestimation of the measured canopy effective volume.

#### 3.1.2 Comparison of EV method predictions with current methods

**Figure 11:**
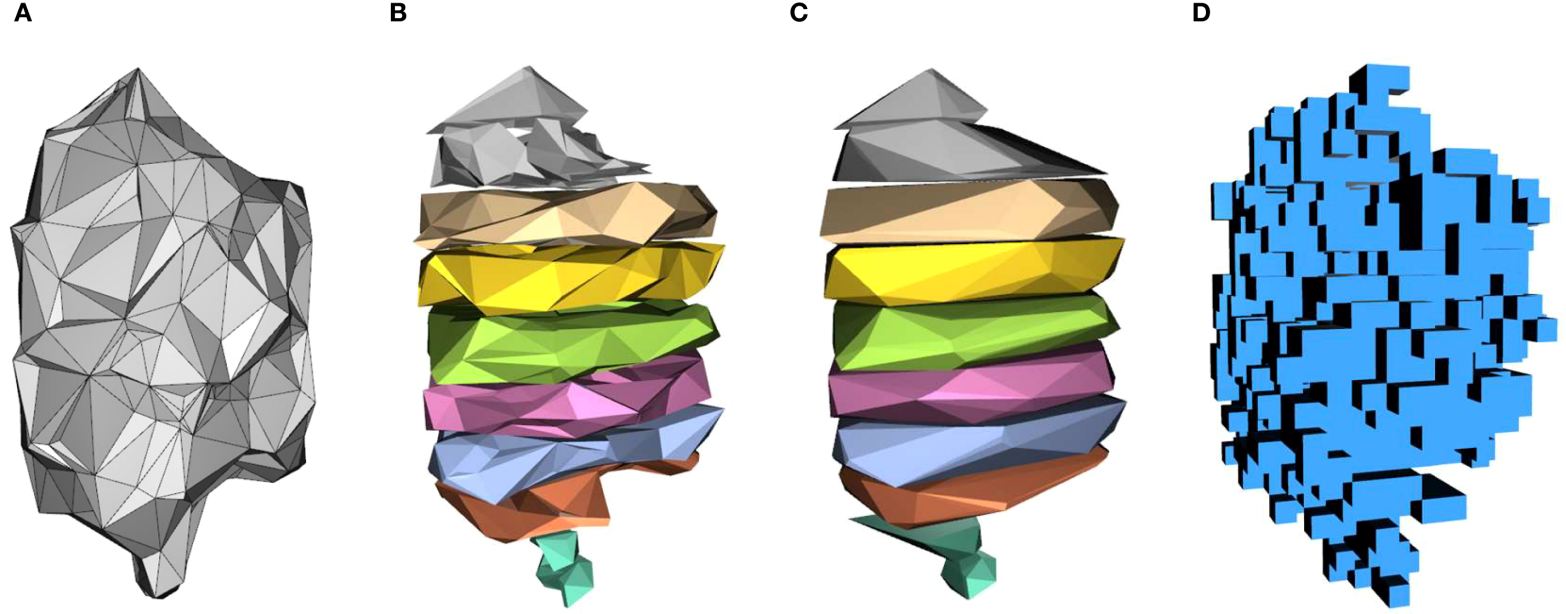
Canopy reconstruction results using different algorithms. **(a)** EV. **(b)** ASBS. **(c)** CHBS. **(d)** VB.

**Figure 12:**
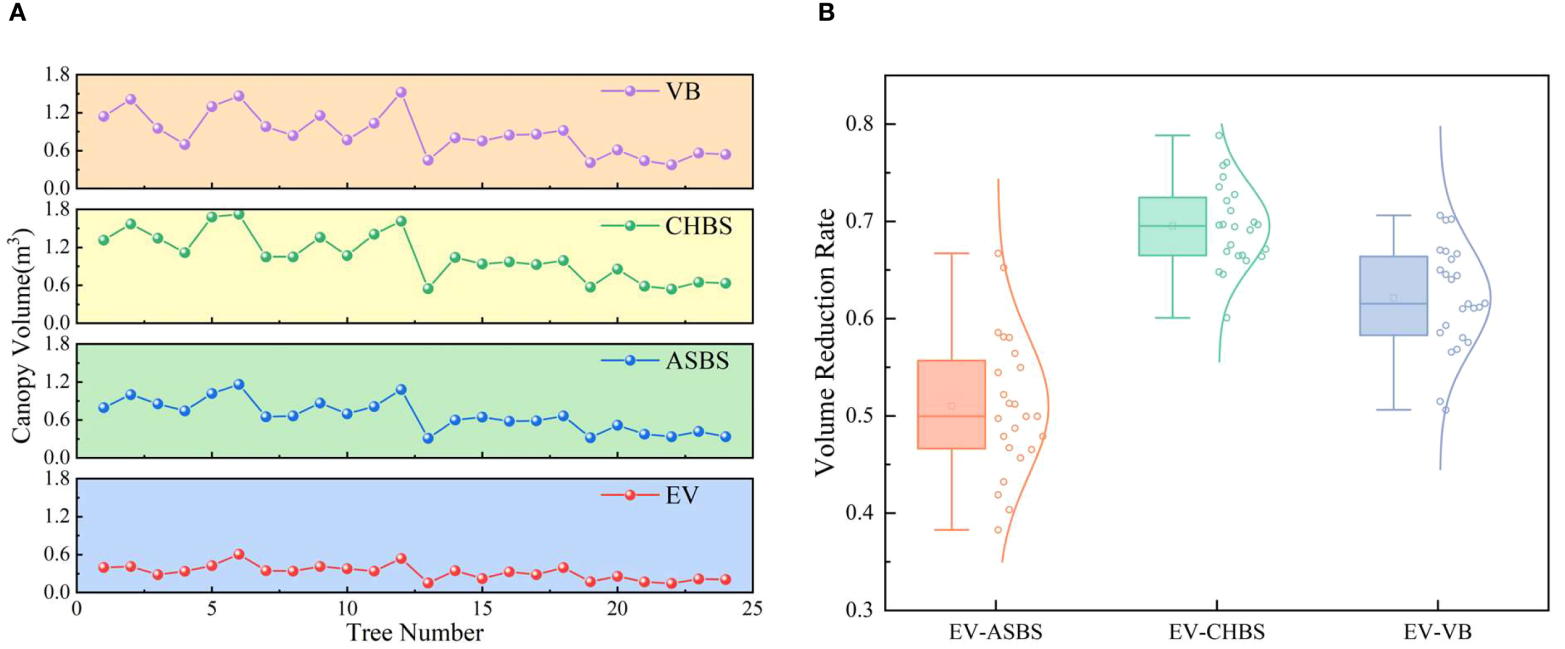
Comparison of predicted results with current methods for simulated fruit trees. **(a)** Comparison of results from different methods. **(b)** Volume reduction rate of the EV method relative to other methods.

In this study, the proposed EV algorithm was compared and analyzed with current canopy volume estimation methods, including the ASBS, CHBS, and VB algorithms. The 3D reconstruction models of the fruit tree canopy, generated using the four algorithms, are illustrated in **Figure 11**, and their corresponding volume calculations are presented in **Figure 12a**. The volume relationship among the methods is as follows: *V_CHBS_* > *V_VB_* > *V_ASBS_* > *V_EV_*.

The CHBS algorithm reconstructs the canopy by slicing the point cloud along the vertical direction and treating each slice as a convex polyhedron (**Figure 11c**). This method encompasses a significant amount of canopy porosity and surface concavity within each convex hull, resulting in a reconstructed model that exceeds the actual canopy boundary and, thus, produces the largest volume estimate among the four methods. The ASBS algorithm also employs a slicing approach but constructs non-convex boundaries for each layer of the point cloud by defining the radius parameter α (**Figure 11b**). This allows the algorithm to capture depressions and some internal porosity on the canopy surface. Since these regions are preserved rather than filled, the estimated volume is relatively lower and closer to the actual volume of the canopy. The VB algorithm divides the 3D space into uniform cubic voxels (**Figure 11d**) and estimates volume by checking whether point cloud data occupies the voxels. While it can identify porosity larger than the voxel edge length, it fails to resolve fine-scale surface depressions or internal porosity smaller than the voxel size. Therefore, its calculation results are intermediate between the CHBS and ASBS algorithms. The EV algorithm first reconstructs the canopy using the AS algorithm (**Figure 11a**), preserving surface depressions, and then applies an effective volume coefficient to remove internal porosity from the reconstructed model. Consequently, the EV algorithm yields the smallest volume estimate, which most closely reflects the accurate effective volume of the canopy.

To evaluate the effectiveness of the EV method in calculating canopy effective volume, the volume reduction rates of its results were computed relative to those of the ASBS, CHBS, and VB methods. The distribution characteristics of these reduction rates are illustrated in **Figure 12b**. The results show that the average volume reduction rates of the EV method compared to the ASBS, CHBS, and VB methods were 0.5101, 0.6953, and 0.6213, respectively. The distribution of the volume reduction rates conformed to a normal distribution as a whole. These results indicate that the EV method has high stability in calculating the canopy effective volume and can effectively remove canopy porosity.

### 3.2 Orchard experiment results and analysis

**Figure 13:**
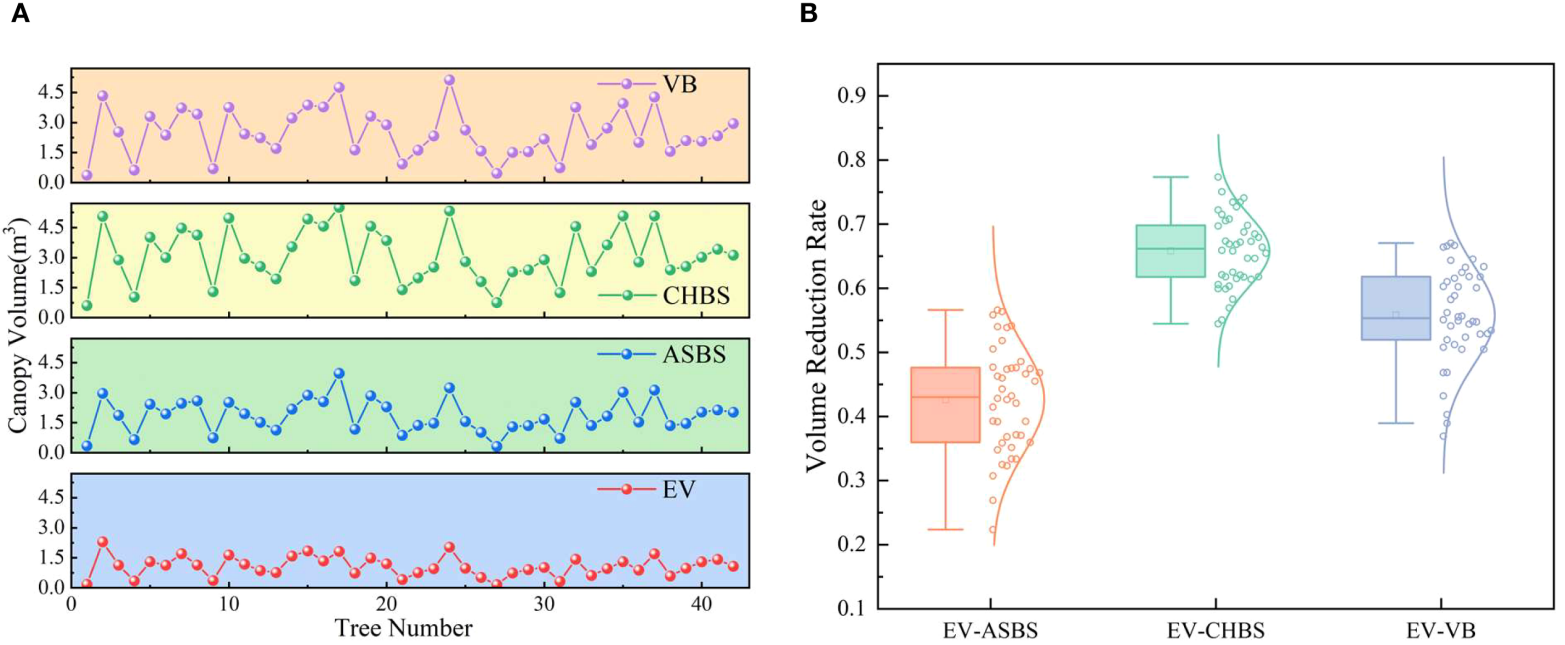
Comparison of predicted results with current methods for fruit trees. **(a)** Comparison of results from different methods. **(b)** Volume reduction rate of the EV method relative to other methods.

The point cloud data in the orchard environment was collected and preprocessed to extract the fruit tree canopy point cloud data in the reconstructed area. Four algorithms, the EV, ASBS, CHBS, and VB methods, were used to calculate the canopy volume, respectively, and the results are presented in **Figure 13a**. The volume relationship measured by the four algorithms is *V_CHBS_* > *V_VB_* > *V_ASBS_* > *V_EV_*, consistent with the results of the simulated fruit tree experiments. Furthermore, the volume reduction rate of the EV algorithm relative to the other three methods was calculated, and the distribution characteristics is shown in **Figure 13b**. The average volume reduction rates of the EV algorithm compared with the ASBS, CHBS, and VB algorithms were 0.4261, 0.6584, and 0.5581, respectively. The distribution of the volume reduction rates also aligns with the results observed in the simulated fruit tree experiments. These findings indicate that the EV algorithm performs well in the complex orchard environment and effectively addresses the overestimation of canopy volume caused by the inclusion of internal porosity in current prediction methods.

### 3.3 Prediction results using different voxel sizes

**Table 1** lists the mean values of canopy effective volume calculated by the EV method, along with the performance evaluation metrics (*R^2^*, *RMSE*, and *MAE*), under different voxel sizes expressed as multiples of the average nearest neighbor distance (*d_avg_*) of the projected point cloud. The result showed that voxel size had a significant impact on the results of canopy effective volume calculation. When the voxel size is set to 0.8×*d_avg_*, the calculated average effective volume is 0.1813, which is only about 40% of the value obtained with a voxel size of 1.2×*d_avg_*. By comparing the results in **Table 2** and meeting the requirement that the predicted value should be less than the measured value, it is evident that the effective volume calculated with a voxel size of 1×*d_avg_* is closest to the measured value. Under this condition, the EV method achieves an *R²* of 0.9720, an *RMSE* of 0.0203 m^3^, and an *MAE* of 0.0191. These results consistently demonstrate that the EV method yields the lowest prediction error and best overall fit when the voxel size is set to 1×*d_avg_*.

**Table 1:** Predicted results for different voxel sizes.

Algorithm	Voxel size	Average of volume predictions	Average of volume measurements	*R^2^*	*RMSE*	*MAE*
EV	0.8×*d_avg_*	0.1813	0.3422	-0.9977	0.1710	0.1609
	0.85×*d_avg_*	0.2151	0.3422	-0.2455	0.1350	0.1271
	0.9×*d_avg_*	0.2508	0.3422	0.3665	0.0963	0.0913
	0.95×*d_avg_*	0.2899	0.3422	0.7902	0.0554	0.0522
	1×*d_avg_*	0.3231	0.3422	0.9720	0.0203	0.0191
	1.05×*d_avg_*	0.3603	0.3422	0.9661	0.0223	0.0184
	1.1×*d_avg_*	0.3962	0.3422	0.7526	0.0602	0.0540
	1.15×*d_avg_*	0.4340	0.3422	0.3401	0.0983	0.0919
	1.2×*d_avg_*	0.4690	0.3422	-0.2795	0.1368	0.1268

**Table 2:** Prediction results for different partition sizes.

Algorithm	Voxel size	Average of volume predictions	Average of volume measurements	*R^2^*	*RMSE*	*MAE*
EV	2×*VS*	0.3892	0.3422	0.8175	0.0517	0.0471
	3×*VS*	0.3715	0.3422	0.9308	0.0318	0.0293
	4×*VS*	0.3588	0.3422	0.9782	0.0179	0.0167
	5×*VS*	0.3231	0.3422	0.9720	0.0203	0.0191
	6×*VS*	0.3108	0.3422	0.9237	0.0334	0.0313
	7×*VS*	0.3006	0.3422	0.8658	0.0443	0.0416
	8×*VS*	0.2940	0.3422	0.8246	0.0507	0.0481
	9×*VS*	0.2856	0.3422	0.7564	0.0597	0.0566
	10×*VS*	0.2805	0.3422	0.7074	0.0654	0.0617

### 3.4 Prediction results using different partition sizes

**Table 2** lists the mean values of canopy effective volume calculated by the EV method, along with the performance evaluation metrics (*R^2^*, *RMSE*, and *MAE*), under different partition sizes expressed as multiples of the voxel sizes(*VS*) of the projected point cloud. The result showed that the partition size also had a significant effect on the effective volume calculation results. By comparing the data in **Table 2** and meeting the requirement that the predicted values should be less than the measured value, it can be determined that the effective volume calculated by the EV method is closest to the measured value when the partition size is set to 5 × *VS*. Under these conditions, the method achieves the lowest prediction error (*RMSE* = 0.0203 m^3^, *MAE* = 0.0191) and demonstrates the best overall stability (*R²* = 0.9720).

## 4 Discussion

Currently, common methods for calculating the canopy volume of fruit trees (such as ASBS, CHBS, and VB algorithms) usually treat the canopy as a solid object and calculate the volume by reconstructing its surface model (Zhu et al., 2021). However, the fruit tree canopy is not a solid body and contains a substantial amount of internal porosity. If the variable spray decision is based on the volume of the canopy containing porosity, it can lead to over-spraying, making it difficult to achieve truly precise spray. In contrast, the EV method proposed in this study effectively addresses the problem of volume overestimation caused by internal porosity in current methods by introducing the canopy effective volume coefficient, which quantifies the influence of porosity on canopy volume calculations.

### 4.1 Discussion on key parameters of the EV method

#### 4.1.1 Voxel size

**Figure 14:**
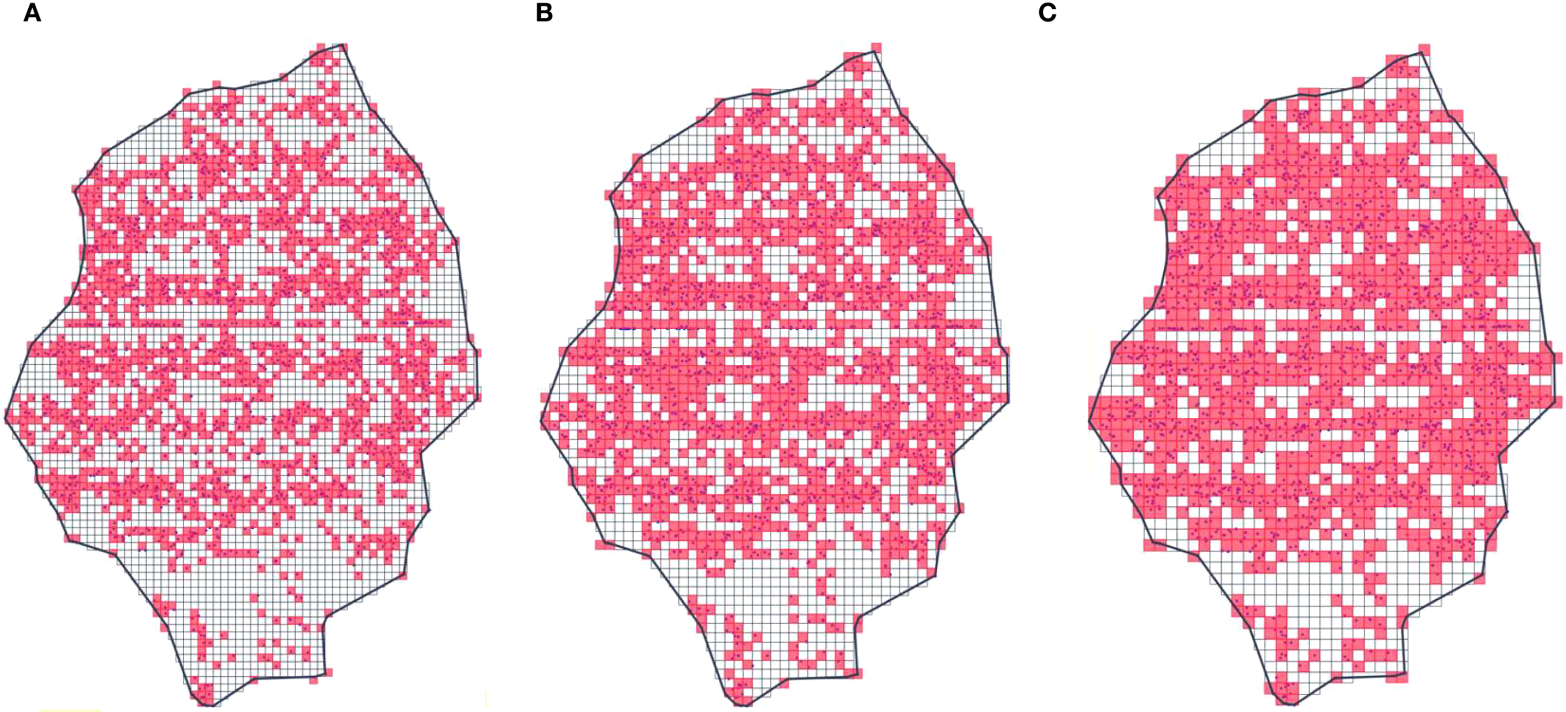
Effect of different voxel sizes. **(a)** 0.8×*d_avg_*. **(b)** 1×*d_avg_*. **(c)** 1.2×*d_avg_*.

**Figure 14** presents the results of processing the fruit tree canopy point cloud data in the yoz projection plane using different voxel sizes. It can be observed that as the voxel size increases, the percentage of the effective voxel area also increases. This phenomenon highlights the critical impact of voxel size selection on the performance of the EV algorithm. When the voxel size is set too small (**Figure 14a**), the effective voxels may fail to fully cover the actual effective canopy area due to the insufficient density of the point cloud data. This leads to an underestimation of the calculated planar effective coefficients, which in turn will result in a small effective volume in the final calculation. Conversely, when the voxel size is set too large (**Figure 14c**), the actual spatial extent represented by a single point cloud data point becomes exaggerated. This causes an overestimation of the planar effective coefficient, ultimately leading to an inflated effective volume calculation.

#### 4.1.2 Partition size

**Figure 15:**
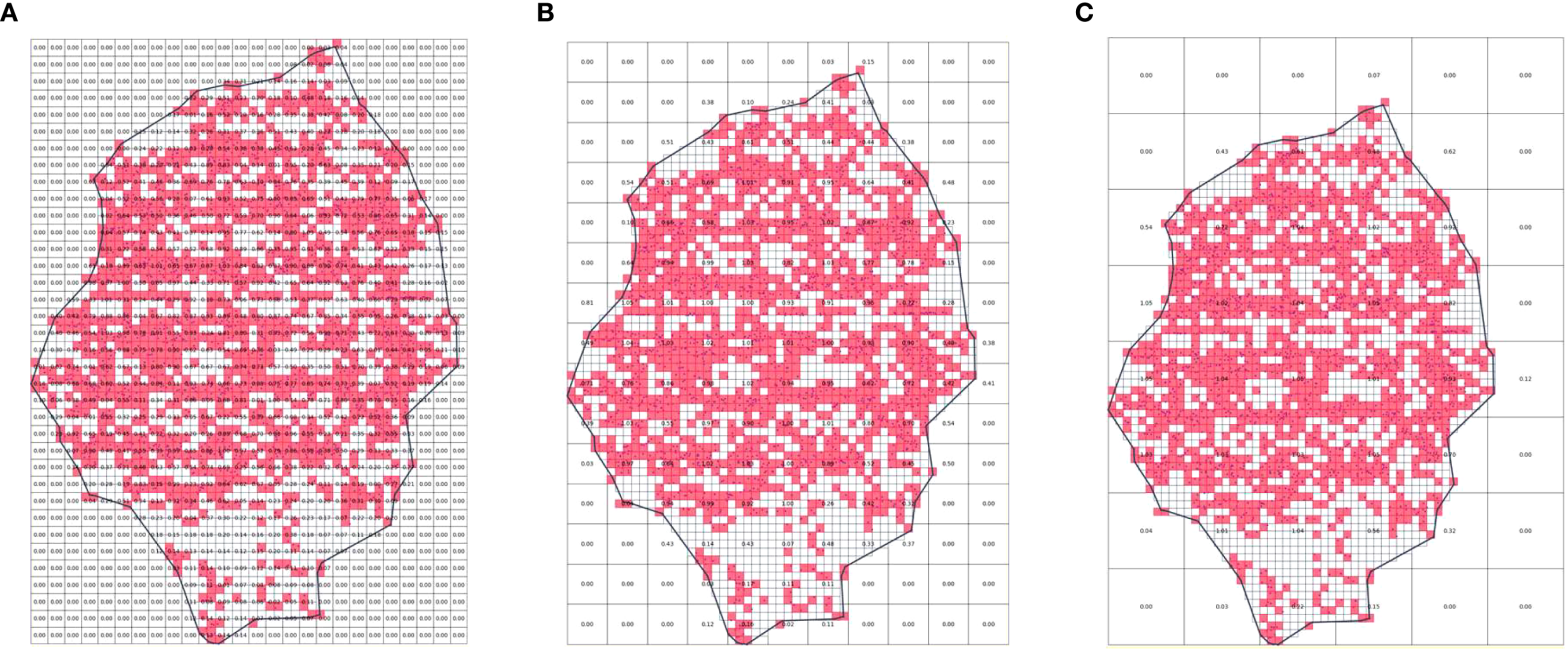
Effect of different partition sizes. **(a)** 2×*VS*. **(b)** 5×*VS*. **(c)** 10×*VS*.

**Figure 15** illustrates the distribution of weights for each partition computed from the fruit tree canopy point cloud data in the yoz projection plane using different partition sizes. The weights are determined by the canopy thickness of the reconstruction model in the projection direction of the corresponding partition. The choice of partition size has a significant impact on the accuracy of weight calculation and the final estimation of canopy effective volume. When the partition size is set too small (**Figure 15a**), the weights obtained from the pointless cloud partitioning calculation tend to be smaller than the actual weights, leading to the overestimation of the planar effective coefficients and, consequently, a calculated effective volume that is too large. Conversely, when the partition size is set too large (**Figure 15c**), each partition usually contains a large amount of point cloud data and covers a wide area. In such cases, some voxels within the partition that lack point cloud data may be assigned higher weights than they should, which results in an underestimation of the planar effective coefficient and, ultimately, a lower calculated canopy effective volume.

### 4.2 The difference in predictive performance between simulated and actual fruit trees for each method

Differences in canopy structure result in different prediction performances between simulated and actual fruit trees for each method. Simulated fruit trees are artificially constructed with dense branches, smaller internal porosity, smoother surfaces, and relatively fewer surface depressions. In contrast, actual fruit trees grow naturally with sparser branches, larger internal porosity, and relatively more surface depressions. The complex, non-convex structure of actual fruit trees is precisely what the ASBS method excels at handling when predicting their volume. Furthermore, during the slicing process, larger porosity sizes within actual fruit trees are better identified and removed, thereby improving the prediction performance of the ASBS method in real fruit trees. When predicting actual fruit trees, the CHBS method contains more surface depressions in its reconstructed model due to the complex, non-convex structure of real trees. However, during the slicing process, internal porosity within the trees is better identified and removed. With the combined effect of these two factors, the prediction performance of the CHBS method also improves for actual fruit trees, though not as significantly as that of the ASBS method. The VB method can only identify porosity larger than its voxel size. The larger internal porosity in actual fruit trees compared to simulated results allows the VB method to better demonstrate its identification capabilities, thereby improving prediction accuracy. the EV method, benefiting from the effective volume coefficient, can precisely quantify the effective volume after removing canopy porosity, resulting in relatively stable prediction performance for both simulated and actual fruit trees.

The results of simulated fruit tree experiments showed that the volume reduction rates for the EV method compared to the ASBS, CHBS, and VB methods were 0.5101, 0.6953, and 0.6213, respectively. In actual orchard experiments, these values were 0.4261, 0.6584, and 0.5581, respectively. The ASBS, CHBS, and VB methods have improved prediction performance in actual fruit trees due to their better handling of large-size porosity, reducing the gap with the EV method’s predictions. This corresponds with our experimental data and indirectly confirms the EV method’s stability when applied to canopy structures containing porosity at different sizes.

### 4.3 Discussion on further research

This study proposes a method for calculating the canopy effective volume of fruit trees. By introducing an effective volume coefficient, it enables precisely quantify the effective volume after removing canopy porosity. However, there are still some shortcomings:

1. This study used the projection method to measure the actual values of canopy effective volume in fruit trees. However, this method is influenced by factors such as the divergent nature of light source, leading to systematic error that causes overestimation of results. Therefore, the results measured by the projection method can serve as a relatively conservative reference standard, but precise measurement methods for actual canopy effective volume still require further research.
2. In this study, when discussing the optimal values of the key parameters voxel size and partition size, the accuracy of the distribution of their values is not refined enough. In future work, more precise optimal parameter values could be determined by combining deep learning with other advanced methods to enhance prediction accuracy further.

## 5 Conclusion

This study aims to accurately quantify the canopy effective volume after removing canopy porosity and provides a method for calculating the canopy effective volume of fruit trees based on LiDAR point cloud data. First, a data preprocessing approach was developed specifically for the standardized tall spindle orchard point cloud data, enabling the effective extraction of fruit tree canopy point clouds and the segmentation of 3D reconstruction areas. Based on this, an alpha-shape canopy reconstruction method based on dynamic parameter optimization was developed for reconstructing canopy regions, and an effective volume coefficient calculation model was constructed. The study analyzed and clarified the influence of core parameters voxel size and partition size on the performance of the method. The method was validated through both simulated fruit tree and orchard experiments. Results demonstrate that the EV method can accurately measure the canopy volume of fruit trees, providing the basis for precise spray technology in orchards. The main conclusions are as follows:

1. A pre-processing procedure of canopy extraction and 3D reconstruction area segmentation for standardized tall spindle orchard point cloud data was developed. An alpha-shape canopy reconstruction method based on dynamic parameter optimization was developed, and the canopy effective volume coefficient was constructed. The effects of voxel size and partition size on the calculation of the effective volume coefficient were analyzed. The optimal parameter values were determined to be the average nearest neighbor distance of the point cloud and five times the voxel size, respectively, based on the density of the canopy point cloud data.
2. The simulated fruit tree experiment results show that the canopy effective volume predicted by the EV method has high accuracy and stability, with evaluation metrics *R²*, *RMSE*, and *MAE* values of 0.9720, 0.0203 m^3^, and 0.0191, respectively. Compared with the prediction results of the ASBS, CHBS, and VB methods, the volume reduction rates were 0.5101, 0.6953, and 0.6213, respectively. The influence of canopy porosity on the volume prediction could be effectively removed. In addition, the orchard experiment results followed a similar trend to the simulated fruit tree experiment results, confirming the method’s applicability in complex orchard environments.

## Data Availability

The raw data supporting the conclusions of this article will be made available by the authors, without undue reservation.
